# Charge transfer in dissociating iodomethane and fluoromethane molecules ionized by intense femtosecond X-ray pulses

**DOI:** 10.1063/1.4944344

**Published:** 2016-03-25

**Authors:** Rebecca Boll, Benjamin Erk, Ryan Coffee, Sebastian Trippel, Thomas Kierspel, Cédric Bomme, John D. Bozek, Mitchell Burkett, Sebastian Carron, Ken R. Ferguson, Lutz Foucar, Jochen Küpper, Tatiana Marchenko, Catalin Miron, Minna Patanen, Timur Osipov, Sebastian Schorb, Marc Simon, Michelle Swiggers, Simone Techert, Kiyoshi Ueda, Christoph Bostedt, Daniel Rolles, Artem Rudenko

**Affiliations:** 1 Deutsches Elektronen–Synchrotron (DESY), 22607 Hamburg, Germany; 2 Max Planck Institute for Nuclear Physics, 69117 Heidelberg, Germany; 3 SLAC National Accelerator Laboratory, Menlo Park, California 94025, USA; 4 Center for Free-Electron Laser Science, DESY, 22607 Hamburg, Germany; 5 Center for Ultrafast Imaging, University of Hamburg, 22761 Hamburg, Germany; 6 J.R. Macdonald Laboratory, Kansas State University, Manhattan, Kansas 66506, USA; 7 Max Planck Institute for Medical Research, 69120 Heidelberg, Germany; 8 Department of Physics, University of Hamburg, 22761 Hamburg, Germany; 9 Sorbonne Universités, UPMC Univ Paris 06, CNRS, UMR 7614, Laboratoire de Chimie Physique–Matiere et Rayonnement, F-75005 Paris, France; 10 Synchrotron SOLEIL, L'Orme des Merisiers, Saint-Aubin, BP 48, F-91192 Gif-sur-Yvette Cedex, France; 11 Extreme Light Infrastructure – Nuclear Physics, Horia Hulubei National Institute for Physics and Nuclear Engineering, RO-077125 Magurele, Jud. Ilfov, Romania; 12 Molecular Materials Research Community, University of Oulu, P.O. Box 3000, FIN-90014 Oulu, Finland; 13 Max Planck Institute for Biophysical Chemistry, 37077 Göttingen, Germany; 14 Institute of X-ray Physics, University of Göttingen, 37077 Göttingen, Germany; 15 IMRAM, Tohoku University, 980-8577 Sendai, Japan; 16 Argonne National Laboratory, Lemont, Illinois 60439, USA; 17 Department of Physics and Astronomy, Northwestern University, Evanston, Illinois 60208, USA

## Abstract

Ultrafast electron transfer in dissociating iodomethane and fluoromethane molecules was studied at the Linac Coherent Light Source free-electron laser using an ultraviolet-pump, X-ray-probe scheme. The results for both molecules are discussed with respect to the nature of their UV excitation and different chemical properties. Signatures of long-distance intramolecular charge transfer are observed for both species, and a quantitative analysis of its distance dependence in iodomethane is carried out for charge states up to I^21+^. The reconstructed critical distances for electron transfer are in good agreement with a classical over-the-barrier model and with an earlier experiment employing a near-infrared pump pulse.

## I. INTRODUCTION

Photo-induced electron transfer plays a central role in a broad range of physical, chemical, and biological reactions, ranging from cometary X-ray emission^1^ to biological light harvesting.^2^ Its microscopic understanding is crucial for emerging photosynthetic,^3,4^ photocatalytic,^5^ and photovoltaic^6,7^ applications. Charge transfer dynamics are governed by concerted electronic and nuclear motion and involve dissipation of electronic energy into the nuclear bath.^8^ Electron transfer phenomena are, therefore, closely related to molecular bond formation and breaking.^9^

In order to experimentally investigate these mechanisms on the atomic level, it is crucial to create a localized charge at a specific atom within a molecule. Using inner-shell ionization followed by (local) Auger decay, multiple charges can be induced with a high degree of spatial localization at a heavy element with a large X-ray absorption cross section. The availability of short-pulsed extreme ultraviolet and X-ray sources, such as high-order harmonics of optical lasers or free-electron lasers (FELs), opened up the way to perform femtosecond time-resolved experiments involving inner-shell electrons, allowing to combine high spatial and temporal resolution. This has recently been exploited to study charge rearrangement processes following multiple core ionization of molecules at the Free-Electron Laser in Hamburg (FLASH),^10,11^ the Linac Coherent Light Source (LCLS),^12,13^ and the SACLA XFEL facility.^14^

The electron dynamics following inner-shell photoabsorption in molecules involve Auger-type relaxation processes and strongly depend on the initial positions of the nuclei and on the interplay between electronic and nuclear motion. Their characteristic time scales are given by the Auger lifetimes, the velocities of the nuclei, and, for multi-photon interactions, by the time delay between the subsequent photoabsorption steps. For molecular fragmentation experiments at FELs in the extreme ultraviolet and X-ray domains,^11–14^ all three time scales are of the order of 1–100 fs, which makes it challenging to trace clear signatures of electron transfer in single-pulse experiments. Recently, internuclear-distance-resolved pump-probe experiments have been performed,^10,15^ where nuclear motion in form of a comparably slow, two-body dissociation reaction is triggered by a laser pump pulse. The relative positions of the nuclei are controlled by the pump-probe delay, allowing the inner-shell ionization to be initiated at a defined internuclear distance.

Two crucial points for this kind of experiments are (*i*) a detailed understanding of the fragmentation induced by the pump pulse, and (*ii*) site-selectivity of the probe–pulse interaction, which creates the initial charge. The early examples of such measurements, such as an XUV-pump, XUV-probe study on I_2_,^10^ a two-color X-ray pump-probe experiment on XeF_2_,^16^ and a near-infrared pump, X-ray probe experiment on CH_3_I,^15^ only partially fulfilled both of these requirements. In particular, in all of the above mentioned experiments, multiple fragmentation channels were populated by the pump pulse, making the relation between the separation of the fragments and the pump-probe delay ambiguous. Ideally, a single dominant dissociation channel should be triggered, allowing for unique reconstruction of the internuclear distance from the delay.

**FIG. 1.**
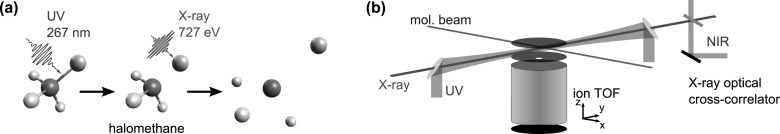
(a) Illustration of the pump-probe experiment. First, the halogen–methyl bond is dissociated by a UV pulse. At a given internuclear distance, the halogen atom is inner-shell ionized by the FEL pulse, creating multiple charges and resulting in fragmentation of the molecule. (b) Sketch of the experimental setup. The UV beam is coupled in collinearly to the X-ray beam via a drilled mirror. In the focus, they are crossed by a supersonic molecular beam. The resulting ionic fragments are imaged by an ion time-of-flight spectrometer. Downstream of the experiment, the arrival time jitter of the FEL is recorded by an X-ray/optical cross-correlator.^29^

In this article, we present the results of a femtosecond pump-probe experiment conducted at the LCLS, aiming at studying electron transfer dynamics following inner-shell ionization of a halogen atom in gas-phase iodomethane (CH_3_I) and fluoromethane (CH_3_F) molecules. The concept of the experiment is schematically illustrated in Fig. 1(a). The molecules are first dissociated by a 267 nm ultraviolet (UV) laser pulse, fragmenting the molecules predominantly into methyl and the halogen atom. The subsequent 1.7 nm (727 eV) X-ray probe pulse mainly ionizes the halogen atom at the iodine (3*d*) or the fluorine (1*s*) shell, respectively, because of their large absorption cross section (3.3 Mb for iodine and 0.39 Mb for fluorine, compared to 0.11 Mb for the methyl group),^17^ resulting in a localized positive charge on the halogen. Depending on the internuclear distance at the time of the X-ray absorption, the charge either remains on the absorbing halogen or spreads over to the molecular environment. By measuring the charge state and the kinetic energy distributions of the created ionic fragments, the charge rearrangement between the two molecular centers can be traced as a function of their internuclear separation.

**FIG. 2.**
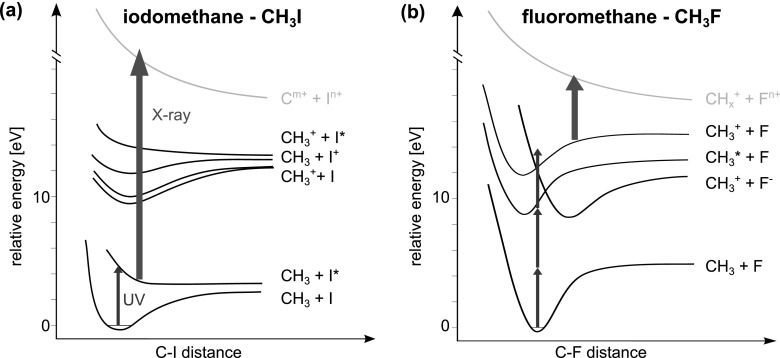
Schematics of relevant potential energy curves for (a) iodomethane and (b) fluoromethane. The energy values are taken from Refs. 39 and 22, respectively. In iodomethane, one UV photon of 267 nm predominantly triggers a resonant neutral dissociation into CH_3_ and I*. The subsequent X-ray ionization at 727 eV populates various highly charged states, the majority of which result in fragmentation of the molecule. Also shown in (a) are ionic curves which were excited by multiphoton near-infrared (NIR)-absorption in Ref. 15. In fluoromethane, more than one UV photon is absorbed, as no resonant states exist, thus exciting several states.

Several considerations motivated the choice of molecular systems and the fragmentation laser wavelength for this experiment. As discussed in our earlier publication,^15^ the iodomethane molecule represents an ideal candidate for this kind of study, because of its efficient two-body break-up and the large difference between the X-ray absorption cross sections of its constituents. However, its multiphoton dissociation by intense 800 nm laser pulses used in Ref. 15 mainly proceeds via ionic channels and yields several pathways of comparable abundance,^18^ which are sketched in the potential curves in Fig. 2(a). In contrast, the 267 nm UV pulse used in this work efficiently triggers a resonant one-photon dissociation into two neutral fragments. The UV-induced photolysis of CH_3_I in the A band is a prototypical photodissociation process and is well studied experimentally and theoretically, see, for example, Refs. 19 and 20 and references therein. The well-known asymptotic kinetic energy of this dissociation allows mapping of the pump-probe delay into internuclear distance, and studying the distance-dependent electron transfer probability.

For fluoromethane, the fragmentation triggered by the UV pulse is more complicated, as illustrated in Fig. 2(b). It mainly proceeds via a singly charged ionic state, with a possible contribution from a neutral ion-pair state.^21,22^ However, CH_3_F is known to exhibit intriguing electron transfer dynamics upon fluorine (1*s*) photoabsorption,^23^ and thus represents an interesting target for charge transfer studies. Moreover, because it is the most electronegative element, fluorine is a predestined candidate to initiate electron transfer from its dissociating molecular environment.

## II. EXPERIMENTAL SETUP

The experiments were performed at the atomic, molecular, and optical physics (AMO) beamline of the LCLS, employing the high-field physics instrument.^24^ As sketched in Fig. 1(b), a collimated, pulsed beam of cold CH_3_I or CH_3_F molecules was crossed with a 267 nm UV pump laser, and an X-ray probe beam in the middle of the ultra-high vacuum reaction chamber. Iodomethane and fluoromethane molecules were delivered to the interaction region using a pulsed Even-Lavie valve (nozzle diameter 150 *μ*m, opening time 10 *μ*s), operated at room temperature. The repetition rate of the LCLS, the pump laser, and the molecular beam was 120 Hz. The liquid CH_3_I sample (vapor pressure at room temperature 540 mbar) was seeded in helium carrier gas (backing pressure 3 bars), the gaseous CH_3_F was expanded directly through the Even-Lavie nozzle (backing pressure 3.5 bars).

The UV and the X-ray beams were propagated collinearly by coupling in the UV beam via a drilled mirror (hole diameter 2 mm) upstream of the interaction region. The LCLS free-electron laser was operated at a photon energy of 727 eV and an average pulse energy of 1 mJ as measured by the LCLS gas monitor detector upstream of the AMO beamline optics.^25^ The transmission of the AMO beamline is estimated to be 15%–20%.^26^ The X-ray beam was focused by a pair of Kirkpatrick–Baez mirrors to a spot size of 3–5 *μ*m^2^. The electron bunch pulse duration of the LCLS was 80 fs for the CH_3_I and 180 fs for the CH_3_F experiment, resulting in an X-ray pulse duration that is typically 60%–70% shorter.^27,28^

The UV pulse had a central wavelength of 267 nm and was created by third harmonic generation driven by an 800 nm Ti:Sapphire laser synchronized with the LCLS. The pulse duration of the UV laser beam was about 100 fs. For the CH_3_F experiment, UV pulses of 95 *μ*J pulse energy were focused onto a spot of 40 *μ*m diameter, whereas for the CH_3_I measurement, 40 *μ*J UV pulses were focused onto a 120 *μ*m diameter spot, resulting in peak intensities of 6 × 10^13 ^W/cm^2^ and 3 × 10^12 ^W/cm^2^, respectively. The UV beam was linearly polarized along the vertical *z*-axis, perpendicular to the polarization direction of the LCLS beam, which was along the *x*-axis, parallel to the molecular beam propagation. Downstream of the experiment, the shot-to-shot arrival time jitter of the FEL with respect to the laser pump pulse was recorded by an X-ray optical cross-correlator,^29^ where the X-ray beam and an optical reference beam with a continuum spectrum are crossed in a silicon nitride sample, and their relative delay is encoded in the spectral profile of the optical probe.

The created ionic fragments were projected along the *z*-axis onto a micro-channel-plate detector by an electrostatic field of an ion time-of-flight (TOF) spectrometer with a round 1 mm^2^ aperture mounted on the extractor electrode, 10 mm from the interaction region. From the recorded time-of-flight spectra, the yields of the respective fragments were calculated, and the kinetic energy distribution corresponding to the momentum component parallel to the spectrometer axis is estimated, following the procedure described in Refs. 30 and 31. On the opposite side of the interaction region, four electron time-of-flight spectrometers were mounted under different angles, and Auger electron spectra were recorded, which are not discussed in the present article. The data analysis was performed with the CFEL–ASG Software Suite (CASS).^32^

## III. RESULTS AND DISCUSSION: IODOMETHANE

**FIG. 3.**
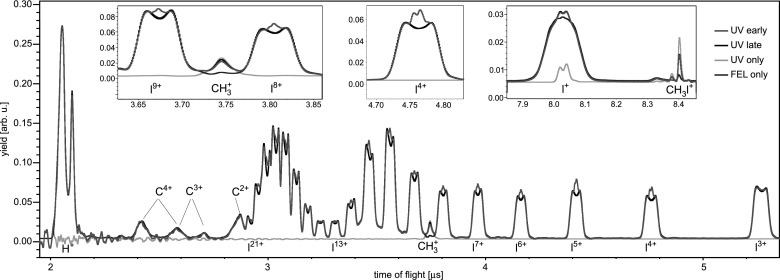
Time-of-flight spectra for iodomethane, resulting from only the UV excitation (green), from only the X-ray ionization (blue), as well as for two different delays between the pulses. “UV early” (red) corresponds to the pump pulse exciting the molecules 170–550 fs before the X-ray ionization, while “UV late” (black) corresponds to the UV pulse arriving 120–730 fs after the X-ray pulse. Note that for the energetic C^3+^ and C^4+^ fragments, the peaks corresponding to ions emitted towards or away from the detector, respectively, overlap with each other, while for C^2+^ the backward peak overlaps with the iodine fragments. The insets show enlarged views for, I^9+^, ${\text{CH}}_{3}^{+}$, I^8+^, I^4+^, I^+^, and CH_3_I^+^ fragments.

The time-of-flight spectra resulting from fragmentation of iodomethane molecules by either the UV pulse or the X-ray pulse, as well as by both pulses at two different delays, are displayed in Fig. 3. The dominant channel triggered with the 267 nm pump pulse, resonant single-photon dissociation into neutral iodine and methyl fragments, is not visible in the ion time-of-flight spectrum. In order to ensure efficient excitation of this channel, and to limit contributions from competing multiphoton pathways, the intensity of the UV pulse was set slightly above the appearance intensity for the ionic fragments. Correspondingly, some singly charged intact molecules as well as ${\text{CH}}_{3}^{+}$ and I^+^ ions are created by the UV pulse. The methyl and iodine fragments both carry little kinetic energy, indicating a dissociation with a neutral partner. The total yield of all ions produced by the UV pulse is very low compared to the fragments created by the X-ray pulse, in spite of the much larger focal volume for the UV pulse.

The ionic fragments produced by the X-ray pulse alone contain highly charged iodine fragments up to I^21+^, as well as carbon ions up to C^4+^, and protons. The high charge states of iodine indicate a large contribution from multi-photon X-ray absorption. For absorption of a single photon at 727 eV in iodomethane, the highest observed charge states are I^5+^ and C^3+^.^33^ Molecular fragments such as CH_3_I^+^ and ${\text{CH}}_{3}^{+}$ are almost absent in the X-ray spectrum, because the iodine (3*d*) inner-shell ionization is followed by cascade Auger decays; thus, multiple charges are created even by a single photon, typically resulting in complete fragmentation of the molecule. The spectrum exhibits a local maximum in the region of the I^16+^–I^21+^ peaks, where the high-energy tails of different charge states overlap, qualitatively resembling the resonant structure observed for xenon atoms at 1500 eV.^31,34^ The I^9+^–I^11+^ peaks most likely contain a contribution from an underlying broad distribution of C^+^, CH^+^, and ${\text{CH}}_{2}^{+}$ ions, while the lower yields of the I^13+^–I^15+^ fragments might be influenced by a reduced detection efficiency of the MCP due to the preceding high signal.

When both pulses interact with the target molecules, several two-color effects can be identified, some of which depend on the delay between pump and probe pulse, as will be discussed below. When comparing the “UV early” and “UV late” spectra to the “FEL only” spectrum in Fig. 3, it can be seen that independent of the delay, the yield of CH_3_I^+^ and ${\text{CH}}_{3}^{+}$ ions is significantly reduced with respect to excitation by only the UV pulse. This is due to target depletion resulting from efficient fragmentation of bound molecules by the X-ray pulse, which were either neutral (“UV late”) or singly charged (“UV early”) before the inner-shell ionization. Note that the “FEL only” spectrum displays lower yields for both species compared to all measurements involving the UV pulse. This is due to the fact that the 267 nm pulse produces significantly more CH_3_I^+^ and ${\text{CH}}_{3}^{+}$ ions than the X-ray pulse, because of the small X-ray valence absorption cross section and the significantly larger focal volume of the UV beam. The observed delay-dependence of the ${\text{CH}}_{3}^{+}$ and CH_3_I^+^ yields is discussed at the end of this section.

The most prominent delay-dependent effect is the appearance of an additional contribution of low-energy iodine ions that arises when the UV pulse excites the molecules before the X-ray ionization. It is visible as a narrow peak in the center of the distributions of each iodine charge state higher than I^3+^, see the enlarged views of the I^4+^, I^8+^, and I^9+^ peaks in the insets of Fig. 3. These ions originate from molecules that are first dissociated into methyl and iodine fragments by the UV pulse and are afterwards locally ionized at the iodine site by the FEL. When the methyl group stays neutral throughout the entire dissociation, the highly charged iodine atoms carry very little kinetic energy. On the other hand, if the methyl is charged while the iodine is still in close proximity, Coulomb repulsion sets in and both fragments are significantly more energetic. In this sense, the yield of highly charged iodine ions with low kinetic energy monitors the delay-dependent probability for the methyl group to remain neutral in the vicinity of a given charge state of iodine, as has been discussed in Ref. 15.

**FIG. 4.**
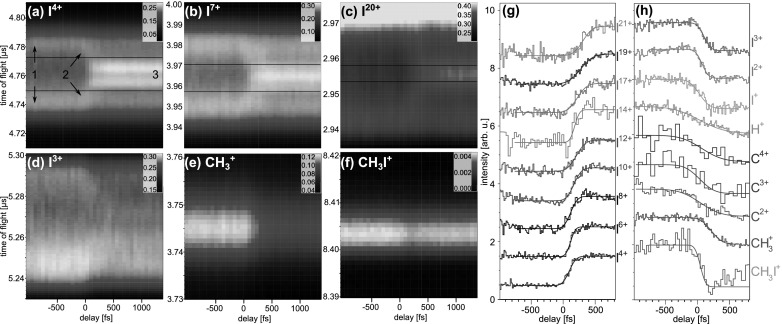
(a)–(f) Time-of-flight spectra as a function of the pump-probe delay for selected fragments of iodomethane. The three different fragmentation channels indicated for I^4+^ fragments in (a) are explained in the text. The pair of black lines marks the region corresponding to channel 3, where the kinetic energy of the fragment is <0.5 eV. In (g), the integrated yield of this low-energy channel is plotted as a function of the delay for several charge states of iodine. Each curve is normalized to a step from zero to one, and they are shifted vertically with respect to each other for better visibility. A Gaussian cumulative distribution function has been fitted to the data. The yields of several other ionic fragments are displayed in (h), each normalized to a step from one to zero. For I^+^, I^2+^, I^3+^, H^+^, ${\text{CH}}_{3}^{+}$, and CH_3_I^+^ ions, the yield integrated over all kinetic energies is shown. For the C^2+^, C^3+^, and C^4+^ peaks, only the non-overlapping regions are considered for the yield in (h), i.e., the forward peaks for C^2+^ and C^4+^, and the backward peak for C^3+^, see Fig. 3.

**FIG. 5.**
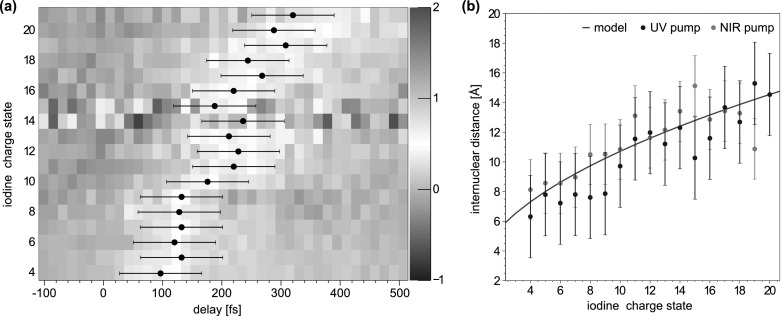
(a) Delay-dependent yields of the low-energy channel for all charge states of iodine. The onset of this channel shifts to larger delays for higher charge states. The mean delay values of the Gaussian cumulative distribution fit functions shown in the 1d-plots in Fig. 4(g) are displayed as black points. (b) Internuclear distances at which the low-energy channel sets in calculated from the pump-probe delay as stated in Eq. (1). Shown are results for UV dissociation from this data set (blue dots), as well as data for NIR dissociation from Ref. 15 (green dots) and calculations from a classical over-the-barrier model^36,37^ (red line). The error bars correspond to the temporal resolutions of 130 and 110 fs for the UV and the NIR experiment, respectively.

The delay-dependent effects are investigated in more detail by plotting the TOF spectra of selected ionic fragments and their respective yields as a function of the pump-probe delay in Fig. 4. For high charge states of iodine, see Figs. 4(a)–4(c), the appearance of low-energy ions at positive delays is clearly visible. The onset of this channel labeled 3 in Fig. 4(a) can be specified by plotting its normalized yield as a function of the delay, as shown in Fig. 4(g) for several charge states. Each curve exhibits a pronounced increase at a given delay that is quantified by fitting it with a Gaussian cumulative distribution function. In Fig. 5(a), the delay-dependent yields of all measured charge states are displayed in a 2d-map, together with the extracted mean values of the Gaussian from the 1d-fit functions. The onset of the low-energy channel 3 clearly shifts to larger delays for higher charge states of iodine. As we have shown previously,^15^ this onset can be used to extract a critical internuclear distance, up to which electron transfer from methyl to iodine is classically allowed for a given charge state.

The dominant fragmentation pathway populated by UV excitation at 267 nm in the present experiment is resonant one-photon excitation of iodomethane to the ^3^Q_0_ state. The asymptotic total energy for the neutral dissociation CH_3_I → CH_3_ + I* at infinite internuclear distance is $E_{\text{kin}}=1.286$ eV,^35^ thus resulting in methyl with 1.15 eV and iodine with 0.136 eV kinetic energy. The internuclear distance *R*(*t*) between the two fragments with masses $m_{{\text{CH}}_{3}}$ and $m_{I}$ can thus be calculated from the pump-probe delay *t* as

$$R\left(t\right)=R_{\text{eq}}+t\sqrt{2E_{\text{kin}}\frac{m_{I}+m_{{\text{CH}}_{3}}}{m_{I}\;m_{{\text{CH}}_{3}}}},$$ (1)

assuming that the dissociating fragments fly apart with constant velocity corresponding to the above asymptotic kinetic energy value. While this assumption is reasonable for large internuclear distances, it neglects the particular shape of the potential surface at small distances and likely overestimates the fragment velocities in that region. The equilibrium distance in the bound molecule is $R_{\text{eq}}$ = 2.2 Å. Figure 5(b) shows as blue dots the distances at which the low-energy channel sets in calculated using Eq. (1) with *t* given by the mean values of the Gaussian cumulative distribution function fits of Fig. 4(g), for all iodine charge states.

**FIG. 6.**
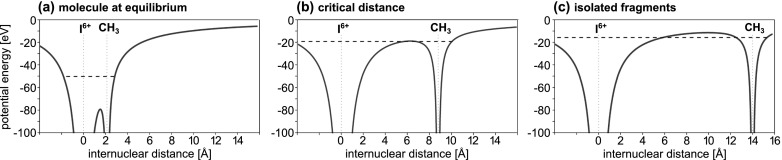
Calculated Coulomb potentials formed by an I^6+^ atom and a neutral methyl group at different internuclear distances. The dashed blue line indicates the energy of the electron in the highest occupied orbital. At the equilibrium distance, (a), the electrons are delocalized over the two sites. When the internuclear distance reaches the critical distance, (b), the potential barrier equals the binding energy of the highest occupied orbital, and for larger distances, (c), the electrons are localized at one of the sites.

The experimental results can be compared to calculated critical distances for classically allowed charge transfer. As was recently shown,^10,15^ a classical over-the-barrier model, developed earlier for slow ion-atom-collisions,^36,37^ can be used to describe the charge redistribution between the fragments of a dissociating molecule. The model is illustrated schematically in Fig. 6. In a bound molecule, at the equilibrium distance, the valence electrons are delocalized over the iodine and the methyl group, see Fig. 6(a). If the nuclei move apart, the Coulombic fields change and the potential barrier between the nuclei rises. At a certain critical internuclear distance, $R_{\text{crit}}$, see Fig. 6(b), the barrier height equals the binding energy of the highest occupied orbital. For larger distances, the electrons can no longer move between the nuclei, but become confined at one of the sites, see Fig. 6(c). Within the model, the critical distance for a given initial charge *q* on the iodine can be calculated as

$$R_{\text{crit}}(q)=\frac{(p+1)+2\sqrt{(p+1)q}}{E_{i}},$$ (2)

in atomic units, where *p* = 0 is the final charge of the methyl group, and *E_i_* = 9.86 eV^38^ is the ionization energy of the least bound valence electron in the CH_3_ radical. In the presence of the Coulomb field created by a charge *q* at a distance *R*, the electron binding energy is then given by $E\prime_{i}=-E_{i}-q/R$ in atomic units, see the dashed blue lines in Fig. 6.

The respective calculated critical distances as a function of the iodine charge state are plotted in Fig. 5(b) as a red line. Moreover, also plotted are distances obtained from a previous NIR-pump, X-ray-probe experiment^15^ as green dots. Within the experimental errors that are dominated by the temporal resolution of 130 fs for the UV data and 110 fs for the IR data, the two data sets agree well with each other, as well as with the predictions of the model. Remarkably, similar values of critical distances are obtained in both experiments, even though the mechanism of NIR-induced dissociation into CH_3_ + I^+^ involves multiphoton absorption, and the resulting fragments are considerably slower as compared to the UV-induced dissociation, with a total kinetic energy of 0.57 eV. This demonstrates that the probability of the electron transfer for a given charge state is essentially defined by the distance between the partners and confirms the adequacy of the classical model^36,37^ within the present resolution limits.

Although the UV excitation at 267 nm initially populates the ^3^Q_0_ state, a small fraction of the dissociating wavepacket can be transferred to the ^1^Q_1_ potential energy surface via nonadiabatic crossing with the ^3^Q_0_ curve.^35^ The reported values for the fraction of this channel with respect to the overall dissociation yield vary from 10% in Ref. 39 to up to 30% in Ref. 40. In the present experiment, this contribution cannot be separated and is therefore neglected in the above conversion of the pump-probe delay into internuclear distance.

Besides the appearance of the low-energy channel 3, other delay-dependent features can be observed in the time-of-flight spectrum. A second dissociative channel labeled 2 can be identified in Figs. 4(a)–4(d), whose kinetic energy rapidly varies as a function of the delay and which connects the high-energy and the low-energy bands at delays between 0 and 500 fs. This feature results from dissociating molecules that undergo Coulomb explosion after the inner-shell ionization, at increasing internuclear distances. In contrast to the low-energy channel, in this case, the dissociation partner is a *charged* methyl fragment, which was either ionized by the UV pulse or through electron transfer to the iodine following the dominant neutral dissociation. The former pathway is limited by the low laser intensity, while the latter becomes prohibitively improbable at larger internuclear distances. Compared to the NIR-dissociation in Ref. 15, this feature is significantly less pronounced in the present data set, due to the dominant neutral fragmentation induced by the UV pulse, as well as the lower energy resolution, and the energy-dependent acceptance of the spectrometer.

Furthermore, for all charge states, the intensity of iodine ions with high kinetic energy in channel 1 is reduced when the UV pulse precedes the X-ray pulse. Since these ions originate from Coulomb explosion of bound molecules, this decrease can be attributed to efficient dissociation of the molecules by the preceding UV pulse. This clear depletion of CH_3_I target by the UV pulse indicates a high fraction of molecules that are dissociated resonantly by absorption of one photon, a process that is significantly more efficient than the multiphoton NIR excitation that was used in Ref. 15. However, it should be noted that the relative strengths of the high- and low-energy channels cannot be directly compared, since the limited acceptance of high-energy fragments of the spectrometer in the present work strongly favors the latter.

The delay-dependent yields of several other ionic fragments, shown in Figs. 4(d)–4(f) and 4(h), exhibit a decrease when the UV pulse precedes the X-ray pulse, opposite to the behavior of the low-energy iodine ions. The yield of I^+^, I^2+^, and I^3+^ ions drops steeply at small positive delays (40, 80, and 110 fs, respectively). In order for these low charge states to be created efficiently after X-ray ionization, several electrons have to be transferred from the methyl group to the iodine site, since the average charge state created by a single photon of 727 eV in an isolated iodine atom is considerably higher. We are not aware of published data for isolated iodine atoms, but the average charge state of xenon, isoelectronic to I^−^, lies between 4 and 6 for ionization at similar photon energies.^41^ I^+^, I^2+^, and I^3+^ ions from dissociating molecules are thus only created at rather short internuclear distances, because at larger distances, it would be very unlikely to transfer this many electrons from the methyl group, and the iodine charge is higher. This is confirmed by the fast decrease of these channels, as compared to the slower increase of highly charged, low-energy fragments (100–310 fs).

Similarly, the yield of ${\text{CH}}_{3}^{+}$ ions also reflects the probability for charge transfer, but this curve is significantly shifted to larger delays (220 fs) as compared to the low charge states of iodine. As mentioned previously, a singly charged methyl fragment can either be created by ionization from the UV pulse or by transferring an electron to a highly charged iodine. However, even though the former mechanism is the dominant one, as can be seen from the comparison of the “UV only” and “FEL only” data in Fig. 3, only the latter pathway results in a significant delay-dependence. The creation of a ${\text{CH}}_{3}^{+}$ fragment via charge transfer requires the transition of exactly one electron, which results in a larger critical distance as compared to the creation of I^+^, I^2+^, and I^3+^, for which several electrons need to be transferred. A direct absorption of an X-ray photon by the charged methyl fragment would result in further break-up and might contribute to the overall reduction of the ${\text{CH}}_{3}^{+}$ signal at positive delays, even though the cross section is small.

The proton yield on the other hand exhibits a very slow decay centered around a delay of 40 fs. Since protons can originate from any fragmentation channel involving break-up of the methyl group, this curve reflects the probability for charge transfer integrated over all iodine charge states and for different numbers of electrons being transferred. Moreover, beyond the dominant dissociation into two neutral fragments, the pump pulse also includes the channels ${\text{CH}}_{3}^{+}$ + I, CH_3_ + I^+^, and CH_2_I^+ ^+ H, see the “UV only” spectrum in Fig. 3. The critical distances for charge transfer to different iodine charge states as well as the velocities of the fragments for different dissociation pathways vary significantly; thus, the proton yield curve exhibits a very broad delay dependence. At negative delays, the UV pulse may post-ionize highly excited molecular fragments created by the FEL pulse, thus increasing the proton yield.

Surprisingly, the yields of C^2+^, C^3+^, and C^4+^ ions also show a broad delay dependence, in stark contrast to the sharp decay of the C^3+^ and C^4+^ signals observed in Ref. 15. Most likely, the reason for this difference is the larger X-ray absorption cross section for carbon fragments in the present experiment at 727 eV, as opposed to 1500 eV. Combined with the smaller X-ray focus of the HFP instrument used here, as compared to the CAMP end station used in Ref. 15, this means that the absorption at carbon is no longer negligible. The initial charge state of the carbon fragment thus becomes undefined to a certain extent, and the yield is averaged over different numbers of electrons transferred to the iodine site, causing a flattening of the delay-dependent carbon ion yield curves.

Finally, the delay-dependence of the CH_3_I^+^ parent ion yield is defined by a small fraction of events, where an X-ray photon was absorbed in a valence shell, leaving the molecule intact. If the X-ray pulse is preceded by the UV pulse, a considerable fraction of the molecules is dissociated, thus reducing the number of bound molecules.

## IV. RESULTS AND DISCUSSION: FLUOROMETHANE

As a complementary study, charge transfer in dissociating fluoromethane molecules was investigated using the same experimental setup. As sketched in Fig. 2(b), resonant one-photon dissociation at 267 nm is not possible in the case of fluoromethane, and the dominant fragmentation originates from dissociative ionization. This process requires the absorption of at least three UV photons; therefore, the UV intensity was chosen about 20 times higher as compared to the iodomethane experiment, in order to achieve a comparable pump-probe contrast. Other pathways that are accessible upon three-photon excitation include the neutral dissociation involving an excited methyl group, as well as the population of the ${\text{CH}}_{3}^{+}+F^{-}$ ion pair dissociation.^22^

**FIG. 7.**
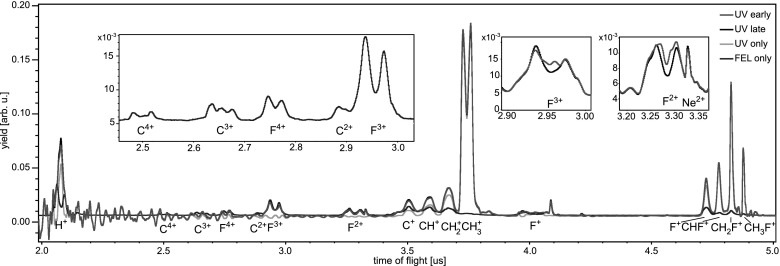
Time-of-flight spectra for fluoromethane resulting from only the UV excitation (green), from only the X-ray ionization (blue), as well as for two different delays between the pulses. “UV early” (red) corresponds to the case where the pump pulse excites the molecules 630–1160 fs before the X-ray ionization, while “UV late” (black) corresponds to the UV pulse arriving 420–1300 fs after the X-ray pulse. The insets show enlarged views for F^3+^ and F^2+^ fragments, as well as high charge states of fluorine and carbon created by the X-ray pulse. The ringing-like noise that is visible in all spectra involving the UV pulse stems from a large contribution of stray light early in the spectrum, caused by the increased UV intensity as compared to the data in Sec. III.

As can be seen in the time-of-flight spectra in Fig. 7, UV excitation mainly results in ${\text{CH}}_{3}^{+}$ fragments, as well as in ${\text{CH}}_{x}^{+}$ and ${\text{CH}}_{x}F^{+}$ ions with x = 0, 1, 2, 3, and protons. A very small contribution from F^+^ ions is also detected. Inner-shell absorption of a single 727 eV X-ray photon and subsequent Auger decay typically creates doubly or triply charged molecules, which then rapidly break up, producing various smaller fragments. Consequently, the spectrum created by the X-ray pulse contains a large proton peak, ${\text{CH}}_{x}^{+}$ fragments, F^2+^ and F^3+^ ions, and a small amount of F^+^, F^4+^, C^2+^, C^3+^, and C^4+^. By comparing this spectrum with single-photon synchrotron data recorded at the same photon energy,^33^ it can be concluded that the F^3+^ and F^4+^ ions, as well as most of the F^2+^, C^2+^, C^3+^, and C^4+^ ions, are produced by multi-photon X-ray absorption.

In a bound molecule, the spreading of the initially localized charge to the molecular environment prevents the creation of fluorine charge states higher than F^2+^. However, those can be produced via absorption of a second X-ray photon at larger internuclear separations, where the charge created locally at the fluorine can no longer be neutralized by electron transfer from the methyl site. We have demonstrated previously that upon X-ray multi-photon ionization, the bond lengths of a small polyatomic molecule can stretch significantly within only 10 fs.^12^ In the present experiment, the FEL pulse duration was 180 fs; thus, the second absorbed X-ray photon most likely interacts with an almost isolated fluorine atom or ion. Calculated F(1*s*) binding energies as obtained from the XATOM program^42^ are F: −687.62 eV, F^+^: −710.51 eV, and F^2+^: −738.50 eV. The photon energy of 727 eV is thus high enough to directly ionize the (1*s*) electron in an isolated F^+^ ion, but not in F^2+^ or higher charge states. Correspondingly, the most abundant fluorine charge state, F^3+^, is predominantly produced by (1*s*) ionization of F^+^, followed by Auger decay, whereas F^4+^ is created either by secondary processes, for example, double Auger decay, or via double-core hole formation in F^+^.

Also shown in Fig. 7 are spectra obtained for two different delays between the UV pump and the X-ray probe pulse. In contrast to the CH_3_I experiment discussed in Sec. III, here the total yield of ions produced by the X-ray pulse is considerably smaller than the ion yield resulting from the UV irradiation, and the spectra are therefore dominated by the fragments produced by the UV pulse. The enlarged views of the time-of-flight traces for F^2+^ and F^3+^ fragments reveal an increased yield of ions in the center (the dip) of the peak for the “UV early” case.

**FIG. 8.**
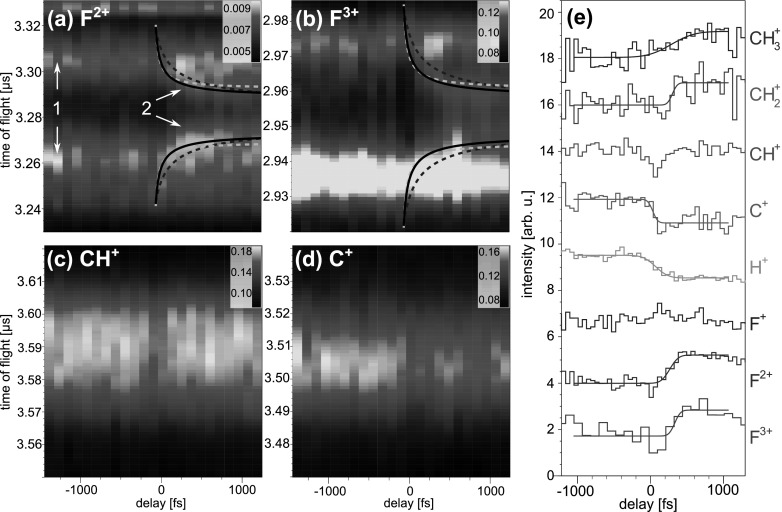
(a)–(d) Time-of-flight spectra as a function of the pump-probe delay for selected fragments of fluoromethane. The two different fragmentation channels indicated for F^2+^ fragments in (a) are explained in the text. Additionally, in (a) and (b), calculated delay-dependent time-of-flight curves are overlayed with the data. They correspond to asymptotic kinetic energies of F^2+^ and F^3+^ of 0.4 eV (black), 0.05 eV (dashed blue), and 0.84 eV (dashed cyan). In (e), the integrated yields of several fragments are plotted as a function of delay. The F^3+^, F^2+^, ${\text{CH}}_{2}^{+}$, and ${\text{CH}}_{3}^{+}$ curves are normalized to show a step from zero to one, and the H^+^ and C^+^ curves to a step from one to zero, and they are shifted vertically with respect to each other for better visibility. A Gaussian cumulative distribution function has been fitted to the data.

The observed delay-dependence is illustrated in more detail by plotting the time-of-flight spectra of selected fragments and their yields as a function of the delay between the UV and the X-ray pulse in Fig. 8. In doubly charged fluorine, two different fragmentation channels are visible, corresponding to pathways 1 and 2 as discussed in Sec. III for iodomethane. Channel 1 has a high kinetic energy that is independent of the delay, whereas the energy of channel 2 varies as a function of delay. In triply charged fluorine, the two analogous channels are clearly distinguishable. The delay-dependent channel 2 corresponds to molecules that are first dissociated by the UV pulse and afterwards inner-shell ionized at the fluorine site by the X-ray pulse. With increasing internuclear distance between the fluorine and its dissociation partner at the time of the X-ray ionization, the kinetic energy of the resulting fluorine fragment decreases because of the smaller Coulomb repulsion. Since ${\text{CH}}_{3}^{+}$ is the most abundant fragment created by the UV pulse, see Fig. 7, it is also the most likely partner of fluorine in the dissociation.

In Figs. 8(a) and 8(b), the results of a Coulomb explosion simulation are shown as overlayed lines. The simulation assumes that the dissociating fragments fly apart with constant velocity corresponding to their asymptotic kinetic energy value, starting at the equilibrium distance at zero time delay. The delay-dependent kinetic energy of the fragments is calculated as a sum of this asymptotic energy and the Coulomb energy,^43^ which is the inverse of the internuclear distance given by Eq. (1), adapted for CH_3_F. The energies are then converted into time of flight by accounting for the geometry of the spectrometer. The asymptotic energies of the fluorine ions are chosen as (*i*) 0.4 eV (black curve), (*ii*) 0.84 eV (dashed cyan), and (*iii*) 0.05 eV (dashed blue), in accordance with (*i*) the mean kinetic energy of the ${\text{CH}}_{3}^{+}$ fragments measured in this work, (*ii*) the maximum of the energy distribution reported for single-photon fragmentation at 18.5 eV photon energy^44^ (∼four 266 nm photons), and (*iii*) the energy for ion pair production^22^ that has a maximum cross section at photon energies around 13–14 eV^45^ (∼three 266 nm photons). Negative fluorine can be produced by excitation to the 3A_1_ and 4A_1_ superexcited states, followed by internal conversion to the 2A_1_ state via avoided crossings, resulting in dissociation into the ion pair ${\text{CH}}_{3}^{+}$ + F^−^.^21^ Although the curves reflect the overall trend observed in the data, neither of them fit the data perfectly, thus not allowing to identify the precursor channel unambiguously within the limitation of this simple Coulomb explosion model.

For fluoromethane, no low-energy channel with a kinetic energy *independent* of the delay is observed in the fluorine ions, as opposed to the iodine ions in iodomethane. In CH_3_I, a resonant single-photon neutral dissociation is accessible with one 267 nm photon, while for CH_3_F, multi-photon dissociative ionization is the dominant process. Because of the large electronegativity of fluorine, the methyl group has a very low probability to remain neutral during the dissociation from the ionic states. This is confirmed by the negligibly small amount of positive fluorine ions in the “UV only” spectrum in Fig. 7. This leads to the conclusion that the main fragmentation pathways triggered by the UV pulse involve the production of ${\text{CH}}_{3}^{+}$, together with either a neutral fluorine atom or its negative ion, which is confirmed by the reasonable agreement of the simulated delay-dependent curves with the experimental data for channel 2.

Similar to the case of iodomethane, the delay-dependent yields in different fragments of fluoromethane plotted in Fig. 8(e) can be interpreted in terms of charge transfer as a function of internuclear distance. If fluorine and methyl are separated further than a given critical distance at the time of the X-ray ionization, the charge created at the fluorine can no longer be neutralized by electron transfer from the methyl site, but remains on the fluorine, thus increasing the yield of F^2+^ and F^3+^ ions. The number of detected F^+^ ions is very low, individual channels are difficult to distinguish, and no clear delay-dependence is observed.

In the carbon-containing fragments, only one channel can be identified. The yields of ${\text{CH}}_{2}^{+}$ and ${\text{CH}}_{3}^{+}$ ions increase if the UV pulse precedes the X-ray pulse, which can be understood when taking into account that the X-ray inner-shell ionization of an intact molecule usually results in fragmentation into several smaller constituents, thus yielding mostly protons and singly charged carbon ions. However, if the molecule is first dissociated by the UV pulse, the X-ray absorption at the fluorine site does not break up the ${\text{CH}}_{2}^{+}$ and ${\text{CH}}_{3}^{+}$ fragments further, once a critical internuclear distance is exceeded. Consequently, the yields of C^+^ and H^+^ ions show the opposite trend, since they are more likely produced when electron transfer to the fluorine atom is still possible. For CH^+^ fragments, both mechanisms balance each other, resulting in a delay-independent yield, except for a pronounced dip at zero delay, where UV and X-ray pulses overlap in time. A possible reason for this may be the creation of highly excited states of CH^+^, which have a relaxation time shorter than the FEL pulse duration and which can be further ionized by a single UV photon, leading to its break-up.

The ${\text{CH}}_{3}^{+}$ ion yield in fluoromethane shows a behavior opposite to what is observed for the case of iodomethane discussed in Sec. III. This reflects the fact that for CH_3_F, the vast majority of the ${\text{CH}}_{3}^{+}$ ions are produced by the UV pulse, see Fig. 7, and thus the delay-dependence in this case results from the depletion of neutral target by the FEL. In iodomethane, the UV focus was three times larger, resulting in a much smaller FEL to UV focal volume ratio. Therefore, the depletion is negligible in CH_3_I, and the delay-dependence is defined by a small contribution of ${\text{CH}}_{3}^{+}$ ions created by X-ray absorption.

## V. CONCLUSION AND OUTLOOK

Charge transfer dynamics following X-ray inner-shell ionization of iodomethane and fluoromethane molecules dissociated by femtosecond UV pulses have been investigated. The experimental results are complementary, due to the qualitatively different reactions of the two species to UV irradiation, as well as the considerably different electronegativities of the two halogen atoms.

For UV excitation of iodomethane, a dominant two-body neutral dissociation pathway with a well-defined fragment energy allows a reliable reconstruction of the internuclear separation at the time of the X-ray absorption, and studying distance-dependent charge transfer dynamics. Very high iodine charge states up to I^21+^ are produced, which enable mapping the critical internuclear distances for electron transfer for a broad range of ionic potentials. The corresponding critical distances of 6 to 15 Å obtained in this experiment agree well with the predictions of a classical over-the-barrier model, as well as with experimental data obtained using an NIR pump pulse.^15^ The non-ionizing UV fragmentation enabled the observation of signatures of charge transfer in other ionic fragments, including singly to triply charged iodine, which could not be studied in the earlier NIR experiment.

Similar to the data presented in Ref. 15, the delay-dependent yield curves for low-energy iodine fragments, which reflect distance-dependent electron transfer rate, not only shift towards larger delays but also become broader for higher charge states. This broadening is not expected within the classical model, which predicts a step-function-like behavior, with the step function being smeared out by the time resolution of the experiment.^15^ This broadening most likely is caused by the exponential distance-dependence of the charge transfer probability;^46^ however, better temporal resolution would be required to resolve the deviations from the model more clearly.

For fluoromethane, a variety of ionizing dissociative pathways are triggered by the UV excitation, making a quantitative analysis of distance-dependent charge transfer dynamics more problematic. In particular, the low-energy channel in the halogen fragment, which was used for monitoring the charge transfer probability in iodomethane, is missing, because of the absence of a dissociation channel involving a neutral methyl fragment. The dynamics are strongly influenced by the dominance of positively charged methyl created in the UV excitation. Nevertheless, signatures of electron transfer could be identified in fluorine and carbon-containing fragments. In addition, depletion of the neutral target by the FEL pulse is observable in the delay-dependent yields of the hydrocarbon fragments. In the case of CH_3_F, this effect is non-negligible because of the smaller UV focus used in order to achieve the relatively high intensity needed to dissociate this molecule.

Another qualitative difference between the two species is the character of the dominant Auger decay pathways. For iodomethane, the first relaxation steps occur as localized transitions involving the intermediate 4*d* shell,^47^ thus enhancing the initial localization of positive charge at the iodine site. For fluorine (1*s*) ionization, the first Auger decay step already involves valence electrons.^48^ Together with the high electronegativity of fluorine, this results in a much lower degree of charge localization at the fluorine, reflected in the low charge states of fluorine observed after X-ray ionization. Within the present experiment, inter-atomic Auger processes cannot be distinguished from fully local decay, followed by valence charge transfer. This could, however, be achieved with coincident Auger electron spectroscopy.

The results presented here support the experimental concept developed in Refs. 10 and 15. The main limiting factor for deducing the functional dependence of the electron transfer probability on the internuclear separation is the limited temporal resolution. With improved timing stability and shorter pulse durations available at current FEL facilities, the pump-probe technique demonstrated here can be used for imaging various charge transfer processes in molecular systems, for example, as a function of the local chemical environment. Sub-femtosecond high-harmonic generation based sources also represent very promising tools for this kind of studies, as demonstrated by recent pioneering measurements on ultrafast charge migration.^49,50^ Combined with coincident electron spectroscopy and upcoming attosecond soft X-ray pulses, our approach will allow tracing local charge propagation dynamics with Ångstrom spatial and sub-femtosecond temporal resolution.
